# Three-Dimensional Cardiac Imaging: A Necessity to the Diagnosis and Treatment of Swiss-Cheese Atrial Septal Defect

**DOI:** 10.7759/cureus.40341

**Published:** 2023-06-12

**Authors:** Andrew S Kao, Shaun Cardozo

**Affiliations:** 1 Internal Medicine, Wayne State University, Detroit, USA; 2 Cardiology, Wayne State University, Detroit, USA

**Keywords:** atrial septal defect (asd) diagnosis, atrial septal defect, percutaneous transcatheter repair, cardiology, three-dimensional echocardiography

## Abstract

Swiss-cheese atrial septal defect (ASD) is a malformation characterized by multi-fenestrated interatrial defects. Here, we describe a vignette of a 23-year-old man with Swiss-Cheese ASD characterized by two defects with areas of 0.74 cm^2^ and 0.44 cm^2^, complicated with an atrial septal aneurysm successfully repaired with a cribriform amplatzer septal occluder (ASO) via the percutaneous transcatheter approach. This case emphasizes the importance of attaining a clear view of three-dimensional structures for proper device selection and deployment in repair as additional structural defects such as concomitant aneurysms impose significant challenges.

## Introduction

Swiss-cheese atrial septal defect (ASD) is a structural finding of the heart used to characterize multiple or fenestrated interatrial defects found in approximately 10% of patients with ASD [1]. The presence of an aneurysm in the atrial septum is an additional malformation associated with fenestrated ASD that further increases the risk of arrhythmia and thromboembolic events [1-2]. Detailed anatomy of the interatrial septum through two- and three-dimensional imaging serves as diagnostic and therapeutic guidance [3]. Current available treatment alternatives include a percutaneous transcatheter approach and open surgical repair. The open surgical repair exhibits similar efficacy but is associated with more complications and is only preferable in the setting of ostium primum, sinus venosus, and coronary sinus defects [4].

## Case presentation

**Figure 1:**
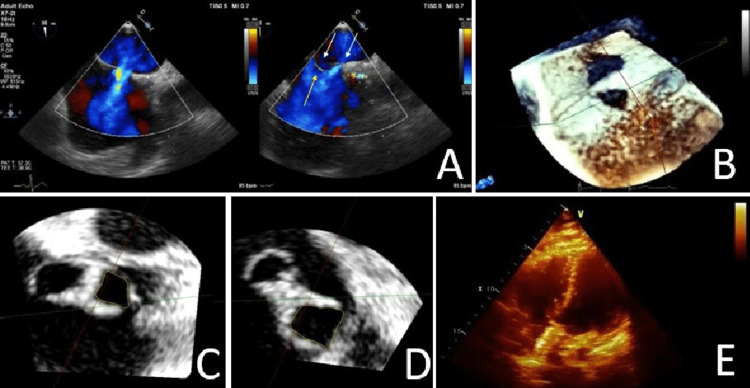
Echocardiography images 1A. Two-dimensional TTE with Doppler demonstrated two interatrial septal defects (white arrows) and a septal aneurysm (yellow arrow). 1B. Three-dimensional TEE demonstrated two interatrial septal defects. 1C. Three-dimensional TEE demonstrated one ASD measured at 0.44 cm^2^. 1D. Three-dimensional TEE demonstrated one ASD measured at 0.74 cm^2^. 1E. Post-procedural TTE demonstrated stable positioned occluder and septum.

A 23-year-old man with a history of malignant migraine and two prior episodes of transient ischemic attacks presented for an elective closure of his suspected patent foramen ovale based on a prior bubble study and negative neurological workup. A prior transthoracic echocardiogram (TTE) revealed an interatrial shunt based on a positive bubble study, with right atrial enlargement, and mild tricuspid regurgitation. A subsequent two-dimensional transoesophageal echocardiogram (TEE) confirmed a left to right shunt with two ASDs and an atrial septal aneurysm (Figure 1A). A three-dimensional TEE confirmed these findings and measured the two interatrial defects with areas of 0.74 cm^2^ and 0.44 cm^2^ (Figure 1B-D). The patient subsequently underwent successful placement of a 35-mm cribriform amplatzer septal occluder (ASO) closure device. TTE at a two-month follow-up revealed a well-positioned closure device without observed shunting (Figure 1E).

## Discussion

A clear three-dimensional analysis of these structures is crucial for the proper deployment of ASO and Gore Helex Septal Occluder, which are safe and effective percutaneous transcatheter devices used in the closure of fenestrated ASDs [5]. ASO is a widely used device comprised of heavy metallic structures on both sides that permanently fuse with the atrial septal wall, whereas the Gore Helex Septal Occluder is softer with less metallic content that can close a defect up to 17 mm in diameter [6]. However, the percutaneous transcatheter approach with either device is challenging with additional structural limitations of an atrial septal aneurysm, deficient ASD rims, and long inter-defect distances [1]. The atrial septal aneurysm may not provide sufficient rims for implantation of standardized ASD occluder, and, thus, complete closure without residual shunts is difficult to achieve without the use of multiple devices [1]. Current literature supports that a single cribriform device is preferred as the large discs stabilize the aneurysmal septum, and the device itself does not rely on the septum for position stability [2,7]. Cribriform ASO device is available in 18, 25, or 35 mm sizes, and its use had successfully repaired 13 out of 16 cases of fenestrated ASD with aneurysmal atrial septum in one study [2]. In comparison to the surgical approach, percutaneous transcatheter repair is less invasive and requires less time for convalescence. Surgery is indicated in the setting of individual preference to avoid foreign device implantation, large ASD defect size, or unfavorable rim structural limitations for any available device [8]. The utility of three-dimensional imaging is that the limited orthogonal-planar view of two-dimensional imaging may fail to reveal additional fenestrations until residual shunts are observed in the post-deployment of a standardized occlude device [9]. Future studies to investigate the disparities of defect size between two- and three-dimensional imaging are warranted. Our case aims to increase clinician’s awareness of Swiss-Cheese ASD complicated by additional structural defects such as the aneurysmal atrial septum, the necessity of a three-dimensional analysis to guide proper device selection, and highlights the promising outcome in closure with the single Cribriform ASO device.

## Conclusions

Swiss-Cheese ASD is an uncommon malformation of multi-fenestrated interatrial defects. Evaluation with three-dimensional echocardiography prior to repair is critical to assess for additional structural defects that can limit proper device deployment. Repairing with the cribriform ASO device via the percutaneous transcatheter approach is an effective method to repair Swiss-Cheese ASD complicated with an atrial septum aneurysm.

## References

[1] Wang Z, Zhan Y, Jin J (2021). Individualized experience with percutaneous transcatheter closure of multiple atrial septal defects: a single-center study. Front Cardiovasc Med.

[2] Numan M, El Sisi A, Tofeig M, Gendi S, Tohami T, El-Said HG (2008). Cribriform amplatzer device closure of fenestrated atrial septal defects: feasibility and technical aspects. Pediatr Cardiol.

[3] Vatankulu MA, Jafarov P, Kahraman Ay N (2015). Swiss cheese-like atrial septal defect detected by three-dimensional echocardiography. Echocardiography.

[4] Warnes CA, Williams RG, Bashore TM (2008). ACC/AHA 2008 guidelines for the management of adults with congenital heart disease: a report of the American College of Cardiology/American Heart Association Task Force on Practice Guidelines (Writing Committee to Develop Guidelines on the Management of Adults With Congenital Heart Disease). Developed in Collaboration With the American Society of Echocardiography, Heart Rhythm Society, International Society for Adult Congenital Heart Disease, Society for Cardiovascular Angiography and Interventions, and Society of Thoracic Surgeons. J Am Coll Cardiol.

[5] Marmagkiolis K, Cilingiroglu M (2014). Have we found the optimal solution for "Swiss cheese" ASDs yet?. Catheter Cardiovasc Interv.

[6] Smith B, Thomson J, Crossland D, Spence MS, Morgan GJ (2014). UK multicenter experience using the Gore septal occluder (GSO(TM) ) for atrial septal defect closure in children and adults. Catheter Cardiovasc Interv.

[7] Tal R, Dahud Q, Lorber A (2013). Fenestrated atrial septal defect percutaneously occluded by a single device: procedural and financial considerations. Cardiol Ther.

[8] Butera G, Biondi-Zoccai G, Sangiorgi G (2011). Percutaneous versus surgical closure of secundum atrial septal defects: a systematic review and meta-analysis of currently available clinical evidence. EuroIntervention.

[9] Looney T, Czaja GR, Flanagan MC, Reoma JL, Hulten E (2018). Case of delayed diagnosis of fenestrated atrial septal defect. Circ Cardiovasc Imaging.

